# Supplementary material for: Association between miRNA Target Sites and Incidence of Primary Osteoarthritis in Women from Volga-Ural Region of Russia: A Case-Control Study
Source: Diagnostics (Basel). 2021 Jul 6;11(7):1222. doi: 10.3390/diagnostics11071222 (PMC8306068; doi:10.3390/diagnostics11071222)
Supplement: Supplementary file 1 [file diagnostics-11-01222-s001.zip › diagnostics-1251473-supplementary.pdf]

Article

Association between miRNA Target Sites and Incidence of Primary Osteoarthritis in Women from Volga-Ural Region of Russia: A Case-Control Study

Anton Tyurin [1,*], Daria Shapovalova [2], Halida Gantseva [1], Valentin Pavlov [3] and Rita Khusainova [4]

1 Internal medicine department, Bashkir State Medical University, 450008 Ufa, Russia; halida.ganceva@mail.ru
2 Laboratory of Human Molecular Genetics, Institute of Biochemistry and Genetics, 450000 Ufa, Russia; daria-ufa92@mail.ru
3 Urology department, Bashkir State Medical University, 450008 Ufa, Russia; pavlov@bashgmu.ru
4 Medical genetics department, Bashkir State Medical University, 450008 Ufa, Russia; ritakh@mail.ru
* Correspondence: anton.bgmu@gmail.com

Table S1. rs9659030 (*COL11A1)*

Comparison Groups	*n*	Allele (%)		Genotype (%)		
		T	C	TT	CT	CC
Controls	148	223 (0.753)	73 (0.247)	78 (0.527)	67 (0.453)	3 (0.02)
Generalized OA	57	98 (0.86)	16 (0.14)	43 (0.754)	12 (0.21)	2 (0.036)
Knee OA	132	200 (0.758)	64 (0.242)	74 (0.561)	52 (0.39)	6 (0.049)
Hip OA	50	82 (0.82)	18 (0.18)	36 (0.72)	10 (0.2)	4 (0.08)
Total OA	242	385 (0.795)	99 (0.205)	155 (0.64)	75 (0.31)	12 (0.05)
Russian with OA	242	385 (0.795)	99 (0.205)	155 (0.64)	75 (0.31)	12 (0.05)
Russian without OA	148	223 (0.753)	73 (0.247)	78 (0.527)	67 (0.453)	3 (0.02)
Tatar with OA	84	126 (0.75)	42 (0.25)	48 (0.571)	30 (0.357)	6 (0. 072)
Tatar without OA	65	94 (0.723)	36 (0. 277)	31 (0.477)	32 (0.492)	2 (0.031)
Mixed and small ethnic groups with OA	69	113 (0.819)	25 (0.181)	49 (0.71)	15 (0.217)	5 (0.073)
Mixed and small ethnic groups without OA	39	63 (0.808)	15 (0.192)	25 (0.641)	13 (0.33)	1 (0.029)

Table S2. rs229069 *(ADAMTS5)*

Comparison Groups	*n*	Allele (%)		Genotype (%)		
		C	G	CC	CG	GG
Controls	161	203 (0.63)	119 (0.37)	72 (0.447)	59 (0.366)	30 (0.187)
Generalized OA	59	80 (0.678)	38 (0.322)	30 (0.508)	20 (0.339)	9 (0.153)
Knee OA	131	186 (0.71)	76 (0.29)	71 (0.542)	44 (0.336)	16 (0.122)
Hip OA	53	78 (0. 736)	28 (0.264)	33 (0.623)	12 (0.226)	8 (0.151)
Total OA	246	349 (0.709)	143 (0.291)	136 (0.553)	77 (0.31)	33 (0.137)

Russian with OA	90	119 (0.66)	61 (0.34)	43 (0.478)	33 (0.367)	14 (0.165)
Russian without OA	49	69 (0.719)	29 (0.281)	24 (0.49)	21 (0.429)	4 (0.081)
Tatar with OA	90	136 (0.756)	44 (0.244)	54 (0.6)	28 (0.311)	8 (0.089)
Tatar without OA	69	94 (0.68)	44 (0.32)	36 (0.522)	22 (0.319)	11 (0.159)
Mixed and small ethnic groups with OA	66	94 (0.712)	38 (0.288)	39 (0.591)	16 (0.242)	11 (0.167)
Mixed and small ethnic groups without OA	43	40 (0.465)	46 (0.535)	12 (0.279)	16 (0.372)	15 (0.349)

Table S3. rs13317 (*FGFR)*

Comparison Groups	*n*	Allele (%)		Genotype (%)		
		T	C	TT	CT	CC
Controls	158	221 (0.699)	95 (0.301)	73 (0.462)	75 (0.475)	10 (0.063)
Generalized OA	61	97 (0.795)	25 (0.205)	40 (0.656)	17 (0.279)	4 (0.065)
Knee OA	139	223 (0.802)	55 (0.198)	88 (0.633)	47 (0.338)	4 (0.029)
Hip OA	53	81 (0.764)	25 (0.236)	32 (0.604)	17 (0.321)	4 (0.075)
Total OA	256	407 (0.795)	105 (0.205)	163 (0.637)	81 (0.316)	12 (0.047)
Russian with OA	95	145 (0.763)	45 (0.237)	58 (0.611)	29 (0.305)	8 (0.084)
Russian without OA	47	66 (0.702)	28 (0.298)	23 (0.489)	20 (0.426)	4 (0.085)
Tatar with OA	90	134 (0.744)	46 (0.256)	48 (0.53)	38 (0.42)	4 (0.05)
Tatar without OA	69	90 (0.652)	48 (0.348)	26 (0.377)	38 (0.551)	5 (0.072)
Mixed and small ethnic groups with OA	71	128 (0.9)	14 (0.1)	57 (0.803)	14 (0.197)	0
Mixed and small ethnic groups without OA	42	65 (0.774)	19 (0.226)	24 (0.571)	17 (0.405)	1 (0.024)

Table S4. rs1061237 (*COL1A1)*

Comparison Groups	*n*	Allele (%)		Genotype (%)		
		T	C	TT	CT	CC
Controls	159	196 (0.616)	122 (0.384)	61 (0.384)	74 (0.465)	24 (0.151)
Generalized OA	61	72 (0.59)	50 (0.41)	22 (0.361)	28 (0.459)	11 (0.18)
Knee OA	135	178 (0.659)	92 (0.341)	61 (0.452)	56 (0.415)	18 (0.133)
Hip OA	50	61 (0.61)	39 (0.39)	18 (0.36)	25 (0.5)	7 (0.14)
Total OA	249	315 (0.633)	183 (0.367)	102 (0.41)	111 (0.446)	36 (0.144)

Russian with OA	94	132 (0.702)	56 (0.298)	47 (0.5)	38 (0.404)	9 (0.096)
Russian without OA	49	55 (0.561)	43 (0.439)	16 (0.327)	23 (0.469)	10 (0.102)
Tatar with OA	88	115 (0.653)	61 (0.347)	40 (0.455)	35 (0.398)	13 (0.147)
Tatar without OA	68	86 (0.632)	50 (0.368)	27 (0.397)	32 (0.471)	9 (0.132)
Mixed and small ethnic groups with OA	67	68 (0.507)	66 (0.493)	15 (0.224)	38 (0.567)	14 (0.209)
Mixed and small ethnic groups without OA	42	55 (0.655)	29 (0.345)	18 (0.429)	19 (0.452)	5 (0.119)

Table S5. rs4647940 *(FGFRL1)*

**Comparison Groups**	***n***	**Allele (%)**		**Genotype (%)**		
		**C**	**G**	**CC**	**GC**	**GG**
Controls	160	289 (0.903)	31 (0.097)	129 (0.806)	31 (0.194)	0
Generalized OA	61	113 (0.926)	9 (0.074)	52 (0.852)	9 (0.148)	0
Knee OA	139	246 (0.885)	32 (0.115)	112 (0.806)	22 (0.158)	5 (0.036)
Hip OA	52	93 (0.894)	11 (0.106)	42 (0.808)	9 (0.173)	1 (0.019)
Total OA	252	452 (0.897)	52 (0.103)	206 (0.817)	40 (0.159)	6 (0.024)
Russian with OA	94	166 (0.883)	22 (0.117)	76 (0.828)	14 (0.142)	4 (0.030)
Russian without OA	49	86 (0.878)	12 (0.122)	37 (0.755)	12 (0.245)	0
Tatar with OA	90	159 (0.883)	21 (0.117)	71 (0.789)	17 (0.189)	2 (0.022)
Tatar without OA	69	126 (0.913)	12 (0.070)	57 (0.859)	12 (0.141)	**0**
Mixed and small ethnic groups with OA	71	132 (0.930)	10 (0.070)	61 (0.859)	10 (0.141)	0
Mixed and small ethnic groups without OA	42	77 (0.917)	7 (0.083)	35 (0.833)	7 (0.167)	0

Table S6. rs5854 *(MMP1)*

**Comparison Groups**	***n***	**Allele (%)**		**Genotype (%)**		
		**C**	**T**	**CC**	**TC**	**TT**
Controls	162	221 (0.682)	103 (0.318)	73 (0.451)	75 (0.463)	14 (0.086)
Generalized OA	61	85 (0.697)	37 (0.303)	33 (0.541)	19 (0.311)	9 (0.148)
Knee OA	135	190 (0.704)	80 (0.296)	66 (0.489)	58 (0.430)	11 (0.081)
Hip OA	53	67 (0.632)	39 (0.368)	20 (0.377)	27 (0.509)	6 (0.113)

Total OA	249	342 (0.687)	156 (0.313)	119 (0.478)	104 (0.418)	26 (0.104)
Russian with OA	94	130 (0.691)	58 (0.309)	43 (0.457)	44 (0.457)	7 (0.740)
Russian without OA	49	63 (0.643)	35 (0.357)	22 (0.449)	19 (0.388)	8 (0.163)
Tatar with OA	87	112 (0.644)	62 (0.365)	37 (0.425)	38 (0.437)	12 (0.138)
Tatar without OA	69	95 (0.688)	43 (0.312)	30 (0.611)	35 (0.296)	4 (0.093)
Mixed and small ethnic groups with OA	54	82 (0.759)	26 (0.241)	33 (0.611)	16 (0.296)	5 (0.093)
Mixed and small ethnic groups without OA	43	61 (0.709)	25 (0.291)	20 (0.465)	21 (0.488)	2 (0.047)

Table S7. rs229077 *(ADAMTS5)*

**Comparison Groups**	***n***	**Allele (%)**		**Genotype (%)**		
		**T**	**C**	**TT**	**TC**	**CC**
Controls	161	128 (0.398)	194 (0.602)	29 (0.180)	70 (0.435)	62 (0.385)
Generalized OA	61	51 (0.418)	71 (0.582)	13 (0.213)	25 (0.410)	23 (0.377)
Knee OA	139	120 (0.432)	158 (0.568)	26 (0.187)	68 (0.489)	45 (0.324)
Hip OA	53	35 (0.330)	71 (0.670)	7 (0.132)	21 (0.396)	25 (0.472)
Total OA	253	206 (0.407)	300 (0.593)	46 (0.182)	114 (0.451)	93 (0.368)
Russian with OA	95	71 (0.374)	119 (0.626)	12 (0.126)	47 (0.495)	36 (0.379)
Russian without OA	84	72 (0.429)	96 (0.571)	16 (0.190)	40 (0.476)	28 (0.333)
Tatar with OA	90	83 (0.461)	97 (0.539)	21 (0.233)	41 (0.456)	28 (0.311)
Tatar without OA	111	94 (0.423)	128 (0.577)	23 (0.207)	48 (0.432)	40 (0.360)
Mixed and small ethnic groups with OA	54	44 (0.407)	64 (0.593)	10 (0.185)	24 (0.444)	20 (0.370)
Mixed and small ethnic groups without OA	65	43 (0.331)	87 (0.669)	8 (0.123)	27 (0.415)	30 (0.462)

Table S8. rs470215 *(MMP13)*

**Comparison Groups**	***n***	**Allele (%)**		**Genotype (%)**		
		**A**	**G**	**AA**	**AG**	**GG**
Controls	129	174 (0.674)	84 (0.326)	57 (0.442)	60 (0.465)	12 (0.093)
Generalized OA	53	74 (0.698)	32 (0.302)	29 (0.547)	16 (0.302)	8 (0.151)
Knee OA	123	173 (0.703)	73 0.297	60 (0.488)	53 (0.431)	10 (0.081
Hip OA	48	60 (0.625)	36 0.375	18 (0.375)	24 (0.500)	6 (0.125)

Total OA	224	307 (0.685)	141 0.315	107 (0.478)	93 (0.415)	24 (0.107)
Russian with OA	87	123 (0.707)	51 0.293	42 (0.483)	39 (0.448)	6 (0.069)
Russian without OA	38	47 (0.618)	29 0.382	16 (0.421)	15 (0.395)	7 (0.184)
Tatar with OA	82	103 (0.628)	61 0.372	34 (0.415)	35 (0.427)	13 (0.159)
Tatar without OA	60	84 (0.700)	36 0.300	27 (0.450)	30 (0.500)	3 (0.050)
Mixed and small ethnic groups with OA	58	86 (0.741)	30 0.259	33 (0.569)	20 (0.345)	5 (0.086)
Mixed and small ethnic groups without OA	31	43 (0.694)	19 0.306	14 (0.452)	15 (0.484)	2 (0.065)

Table S9. rs1042840 *(MMP13)*

Comparison Groups	*n*	Allele (%)		Genotype (%)		
		A	G	AA	AG	GG
Controls	147	204 (0.694)	90 (0.306)	70 (0.476)	64 (0.435)	13 (0.088)
Generalized OA	57	84 (0.737)	30 (0.263)	32 (0.561)	20 (0.351)	5 (0.088)
Knee OA	125	179 (0.716)	71 (0.284)	62 (0.496)	55 (0.440)	8 (0.064)
Hip OA	48	72 (0.750)	24 (0.250)	29 (0.604)	14 (0.292)	5 (0.104)
Total OA	230	335 (0.728)	125 (0.272)	123 (0.535)	89 (0.387)	18 (0.078)
Russian with OA	88	132 (0.750)	44 (0.274)	47 (0.534)	38 (0.381)	3 (0.084)
Russian without OA	45	58 (0.644)	32 (0.356)	18 (0.400)	22 (0.489)	5 (0.111)
Tatar with OA	82	123 (0.750)	41 (0.250)	45 (0.549)	33 (0.402)	4 (0.091)
Tatar without OA	66	93 (0.705)	39 (0.295)	33 (0.500)	27 (0.409)	6 (0.091)
Mixed and small ethnic groups with OA	63	86 (0.683)	40 (0.317)	34 (0.540)	18 (0.286)	11 (0.175)
Mixed and small ethnic groups without OA	36	53 (0.736)	19 (0.264)	19 (0.528)	15 (0.417)	2 (0.056)

Table S10. rs1061947 *(COL1A1)*

Comparison Groups	*n*	Allele (%)		Genotype (%)		
		A	G	AA	GA	GG
Controls	159	68 (0.214)	250 (0.786)	6 (0.038)	56 (0.352)	97 (0.610)
Generalized OA	58	23 (0.198)	93 (0.802)	2 (0.034)	19 (0.328)	37 (0.638)
Knee OA	134	40 (0.149)	228 (0.851)	2 (0.015)	36 (0.269)	96 (0.716)
Hip OA	53	21 (0.198)	85 (0.802)	3 (0.057)	15 (0.283)	35 (0.660)

Total OA	248	85 (0.171)	411 (0.829)	7 (0.028)	71 (0.286)	170 (0.685)
Russian with OA	95	27 (0.142)	163 (0.858)	2 (0.021)	23 (0.242)	70 (0.737)
Russian without OA	49	16 (0.163)	82 (0.837)	1 (0.020)	14 (0.286)	34 (0.694)
Tatar with OA	88	30 (0.170)	146 (0.830)	2 (0.023)	26 (0.295)	60 (0.682)
Tatar without OA	68	29 (0.213)	107 (0.760)	1 (0.058)	27 (0.365)	40 (0.577)
Mixed and small ethnic groups with OA	52	25 (0.240)	79 (0.760)	3 (0.058)	19 (0.365)	30 (0.577)
Mixed and small ethnic groups without OA	42	23 (0.274)	61 (0.726)	4 (0.095)	15 (0.357)	23 (0.548)

Table S11. rs9978597 *(ADAMTS5)*

Comparison Groups	*n*	Allele (%)		Genotype (%)		
		T	G	TT	TG	GG
Controls	155	298 (0.961)	12 (0.039)	145 (0.935)	8 (0.052)	2 (0.013)
Generalized OA	58	110 (0.948)	6 (0.052)	52 (0.897)	6 (0.103)	0
Knee OA	135	252 (0.933)	18 (0.067)	118 (0.874)	16 (0.119)	1 (0.007)
Hip OA	51	97 (0.951)	5 (0.049)	47 (0.922)	3 (0.059)	1 (0.020)
Total OA	247	464 (0.939)	30 (0.061)	219 (0.887)	26 (0.105)	2 (0.008)
Russian with OA	92	171 (0.929)	13 (0.071)	81 (0.880)	9 (0.098)	2 (0.022)
Russian without OA	46	88 (0.957)	4 (0.043)	42 (0.913)	4 (0.087)	0
Tatar with OA	87	165 (0.948)	9 (0.052)	78 (0.897)	9 (0.103)	0
Tatar without OA	66	129 (0.977)	3 (0.023)	64 (0.970)	1 (0.015)	1 (0.015)
Mixed and small ethnic groups with OA	68	128 (0.941)	8 (0.059)	60 (0.882)	8 (0.118)	0
Mixed and small ethnic groups without OA	43	81 (0.942)	5 (0.058)	39 (0.907)	3 (0.070)	1 (0.023)

Table S12. rs73611720*(GDF5)*

Comparison Groups	*n*	Allele (%)		Genotype (%)		
		T	G	TT	TG	GG
Controls	161	281 (0.873)	41 (0.127)	125 (0.776)	31 (0.193)	5 (0.031)
Generalized OA	60	108 (0.900)	12 (0.100)	49 (0.817)	10 (0.167)	1 (0.017)
Knee OA	140	251 (0.896)	29 (0.104)	113 (0.807)	25 (0.179)	2 (0.014)
Hip OA	52	94 (0.904)	10 (0.096)	42 (0.808)	10 (0.192)	0

Total OA	252	453 (0.899)	51 (0.101)	204 (0.810)	45 (0.179)	3 (0.012)
Russian with OA	93	169 (0.909)	17 (0.091)	77 (0.828)	15 (0.161)	1 (0.011)
Russian without OA	49	92 (0.939)	6 (0.061)	44 (0.898)	4 (0.082)	1 (0.020)
Tatar with OA	89	156 (0.876)	22 (0.124)	69 (0.775)	18 (0.202)	2 (0.022)
Tatar without OA	69	122 (0.884)	16 (0.116)	54 (0.783)	14 (0.203)	1 (0.014)
Mixed and small ethnic groups with OA	70	128 (0.914)	12 (0.086)	58 (0.829)	12 (0.171)	0
Mixed and small ethnic groups without OA	43	67 (0.779)	19 (0.221)	27 (0.628)	13 (0.302)	3 (0.070)

Table S13. rs6854081*(FGF2)*

**Comparison Groups**	***n***	**Allele (%)**		**Genotype (%)**		
		**T**	**G**	**TT**	**TG**	**GG**
Controls	144	263 (0.913)	25 (0.087)	121 (0.840)	21 (0.146)	2 (0.014)
Generalized OA	61	107 (0.877)	15 (0.123)	47 (0.770)	13 (0.213)	1 (0.016)
Knee OA	141	258 (0.915)	24 (0.085)	119 (0.844)	20 (0.142)	2 (0.014)
Hip OA	52	89 (0.856)	15 (0.144)	38 (0.731)	13 (0.250)	1 (0.019)
Total OA	254	454 (0.894)	54 (0.106)	204 (0.803)	46 (0.181)	4 (0.016)
Russian with OA	94	167 (0.888)	21 (0.112)	75 (0.798)	17 (0.181)	2 (0.021)
Russian without OA	47	81 (0.862)	13 (0.138)	36 (0.766)	9 (0.191)	2 (0.043)
Tatar with OA	90	151 (0.839)	29 (0.161)	63 (0.700)	25 (0.278)	2 (0.022)
Tatar without OA	56	106 (0.946)	6 (0.054)	50 (0.862)	6 (0.103)	0
Mixed and small ethnic groups with OA	70	136 (0.971)	4 (0.029)	66 (0.943)	4 (0.057)	0
Mixed and small ethnic groups without OA	41	76 (0.927)	6 (0.073)	35 (0.854)	6 (0.146)	0

Table S14. rs1042673*(SOX9)*

**Comparison Groups**	***n***	**Allele (%)**		**Genotype (%)**		
		**A**	**G**	**AA**	**GA**	**GG**
Controls	161	168 (0.522)	154 (0.478)	47 (0.292)	74 (0.460)	40 (0.248)
Generalized OA	61	69 (0.566)	53 (0.434)	19 (0.311)	31 (0.508)	11 (0.181)
Knee OA	139	165 (0.594)	113 (0.406)	50 (0.360)	65 (0.468)	24 (0.172)
Hip OA	52	52 (0.500)	52 (0.500)	13 (0.206)	26 (0.413)	13 (0.381)

Total OA	255	289 (0.567)	221 (0.433)	82 (0.322)	125 (0.490)	48 (0.188)
Russian with OA	94	109 (0.580)	79 (0.420)	28 (0.298)	53 (0.564)	13 (0.138)
Russian without OA	49	57 (0.582)	41 (0.418)	18 (0.346)	21 (0.404)	10 (0.250)
Tatar with OA	90	104 (0.578)	76 (0.422)	32 (0.356)	40 (0.444)	18 (0.200)
Tatar without OA	69	67 (0.486)	71 (0.514)	17 (0.246)	33 (0.478)	19 (0.275)
Mixed and small ethnic groups with OA	71	76 (0.535)	66 (0.465)	22 (0.310)	32 (0.451)	17 (0.239)
Mixed and small ethnic groups without OA	43	44 (0.512)	42 (0.488)	12 (0.279)	20 (0.465)	11 (0.256)
